# Cracking susceptibility of full-sibs of a cross of a cracking tolerant and cracking susceptible sweet cherry: Relation to cuticle characteristics, microcracking and calcium

**DOI:** 10.1371/journal.pone.0316637

**Published:** 2025-01-03

**Authors:** Moritz Knoche, Loise Grosset-Grange, José Quero-García, David Alletru, Lina Boutaleb

**Affiliations:** 1 Institute for Horticultural Production Systems, Leibniz University Hannover, Hannover, Germany; 2 INRAE, Biologie du Fruit et Pathologie, Université de Bordeaux, UMR 1332, Villenave d’Ornon, France; Nuclear Science and Technology Research Institute, ISLAMIC REPUBLIC OF IRAN

## Abstract

Rain cracking compromises quality and quantity of sweet cherries worldwide. Cracking susceptibility differs among genotypes. The objective was to (1) phenotype the progeny of a cross between a tolerant and a susceptible sweet cherry cultivar for cuticle mass per unit area, strain release on cuticle isolation, cuticular microcracking and calcium/dry mass ratio and (2) relate these characteristics to cracking susceptibilities evaluated in laboratory immersion assays and published multiyear field observations. Mass of the dewaxed cuticle per unit area and strain release upon cuticle isolation were significantly related to cracking susceptibility in lab or field. Cuticular microcracking in the stylar end region as indexed by infiltration with acridine orange was more severe in susceptible than in tolerant genotypes and significantly correlated with susceptibility to cracking in lab and field. The Ca/dry mass ratio was lower (-8%) for susceptible than for tolerant genotypes. Fruit that cracked early had less Ca than those that cracked later. Only the Ca/dry mass ratio of the stylar end region was significantly correlated with cracking susceptibility in the field. Based on stepwise regression analyses microcracking of the cuticle accounted for most of the cracking susceptibilities in field and lab (partial r^2^ = 0.331 to 0.338 for field vs. r^2^ = 0.326 to 0.453 for lab). The variability in cracking susceptibility accounted for increased to a r^2^ = 0.571 (lab) when adding mass of dewaxed cuticle, up to r^2^ = 0.421 (field) when adding the Ca/dry mass ratio in the stylar end region or up to r^2^ = 0.478 (field) when entering the strain release on isolation into the model. A protocol for phenotyping is suggested that allows larger progenies to be phenotyped for microcracking, DCM mass and strain release.

## Introduction

Rain cracking of sweet cherry fruit is a limitation in production of this crop in all areas of the world where rain occurs before or during the harvest season. Even low percentages of cracking render a harvest uneconomical due to the increased labor input required for inspection and grading. Furthermore, fruit that was exposed to surface moisture suffers from microscopic cracking [1]. This, in turn, results in unrestricted water transport and possibly shrivel [1, 2] and an increased incidence of fruit rots [3].

Sweet cherry fruit cracking is an active field of research [4–12]. Only recently a coherent model consistent with all experimental data has been published [13, 14]. According to the so-called ‘Zipper’ model, cracking of sweet cherry fruit is the final result of a sequence of events [13, 14]. Cutin and wax deposition ceases early during fruit development at the onset of color change [15, 16]. The subsequent increase in fruit mass then distributes an essentially constant amount of cuticle over an increasing surface area causing significant strain. Strain of the cuticle polymer results in formation of microscopic cracks, the so called microcracking, of the cuticle [17]. As a consequence, the cuticle’s barrier function is impaired. In addition, exposure of the strained cuticle to surface moisture (liquid water) or high humidity aggravates microcracking [1]. Microcracks further impair the cuticle’s barrier function in water movement and pathogen defense. Microcracks focus water uptake into the tissue immediately underlying the microcrack [18], but do not compromise the mechanical properties of the fruit skin [19]. Uptake through microcracks is rapid and occurs by viscous flow. Water is then taken up from the cell wall space into the large thin-walled cells of the outer flesh where the osmotic potential is more negative than in the epidermis and hypodermis of the skin [20]. The cells of the flesh begin to burst [21]. Sweet cherries contain large amounts of malic acid which is subsequently released into the cell wall space. As a result, the permeabilities of the plasma membrane and tonoplast of adjacent cells increase causing even further leakage [22]. The consequences are several fold. First, malic acid extracts calcium from the cell walls resulting in decreased cross linking [23]. Second, cell walls now swell due to hydration and cell-to-cell adhesion decreases [22, 24, 25]. Third, the weakening of cell-to-cell adhesion and the stress concentration that occurs at the tip of microcracks allows microcracks to extend into macrocracks that ‘run’ over the fruit surface and extends deep into the flesh [14]. The Zipper model is consistent with all experimental findings to date and accounts for cracking of sweet cherry fruit in the absence of significant turgor [26, 27].

Sweet cherry fruit cracking is affected by environmental, but also by genetic factors. Among the environmental factors surface moisture is clearly most critical and the exclusion of surface moisture by rain shelters most effective in decreasing cracking. However, rain shelters are expensive and not always easy to install depending on training systems and topography [28–30]. In extreme conditions, they are not sufficiently effective in preventing cracking (Quero-Garcia and Knoche, unpublished data).

From the genetic point of view, it is well established that sweet cherry cultivars differ in their susceptibility to cracking [6, 31, 32]. The genetic basis for this difference is currently unknown. Recently, the first paper that identified QTLs for cracking susceptibility evaluated in segregating populations has been published [33]. The largest and most robust QTLs were reported for stylar end cracking which is the most frequent type of cracking [33, 34]. QTLs for cracking tolerance were also recently reported by GWAS approaches [35, 36], which allowed the identification of new genomic regions involved in cracking susceptibility while confirming the significance of the first QTLs reported by [33]. The molecular basis of these QTLs is still not clear and remains to be identified. In particular information would be helpful on which one or several of the above events of the Zipper model is/are the most critical in determining the cracking susceptibility of a particular genotype. This could help refining the confidence intervals of the detected QTLs or even identifying the underlying candidate genes. From a practical point of view, the gain in the precision of QTL detection would help to improve the design of molecular markers linked to cracking tolerance which could be in turn used by breeders in marker-assisted selection strategies [37].

The objectives of the present study were (1) to phenotype a range of genotypes of sweet cherry fruit for selected key traits identified in the Zipper model and for their susceptibility to cracking and (2) to propose a new phenotyping protocol that could be implemented in larger segregating populations to disentangle the genetic determinants of cracking tolerance. We selected the same segregating population of sweet cherry fruit for which the QTLs for cracking susceptibility were established earlier [33]. We focused on cuticle mass per unit area, strain of the cuticle, microcracking and the fruit calcium relations because these represent critical events in the Zipper model.

## Materials and methods

### Plant materials

Mature sweet cherry fruit (*Prunus avium*) were sampled from a segregating population of 12-year-old trees of sweet cherry. The trees represented full siblings of the cross ‘Regina’ × ‘Garnet’ with two trees per genotype. These parent cultivars differed in cracking susceptibility with ‘Regina’ being cracking tolerant and ‘Garnet’ cracking susceptible. The trees were grafted on MaxMa14 rootstocks (*Prunus mahaleb* × *P*. *avium*) in 2010 and planted in 2012. Trees were grown at the Horticultural Research Station of the INRAE in Toulenne, France (latitude 44.57 N, longitude 0.28 W) according to current regulations for integrated fruit production. The research station is part of the UEA INRAE Experimental Unit and both are part to the Bordeaux-Aquitaine INRAE Center. No foliar fertilizers were applied. Fruit were picked at commercial maturity based on color and size and selected for freedom from defects and uniformity of development. The remaining sample of 110 fruit per genotype was held overnight in cold storage at 2°C for processing on the next day.

### Methods

#### Cracking assessment in the field

In this work, we also used data from 8 years of field observations (from 2008 till 2016) on cracking that were obtained on seedling trees of the same ‘Regina’ × ‘Garnet’ cross as above. The data obtained on these siblings served as the basis for the QTL study carried out earlier [33]. The trees were grown at the same site in a block adjacent to the grafted trees that served as a source of fruit for the present study. Thus, the genotype and the growing environment (soil, (micro)climate), but not the rootstock and the growing seasons, were identical in the earlier and the present study. The following data sets from [33] were used: the mean percentage of fruit per genotype and per year that exhibited cracking in the stylar end region (‘stylar end cracking’), the mean percentage of fruit per genotype and per year that cracked regardless of the region of cracking (‘maximum cracking’) and the predicted percentage of cracking in the stylar end region that is corrected for rain fall in the respective growing season and for fruit mass and firmness (‘predicted cracking’). For further details the reader is referred to [33].

#### Laboratory cracking assay

The cracking susceptibility of fruit from the grafted siblings was quantified in 2022 using a standardized laboratory-based incubation assay [38, 39] that we modified. Briefly, pedicels were cut to standard length of 1 cm using a razor blade. Fruit were graded for equal mass into three groups of 25 fruit each (for mean mass data per genotype see S1 Dataset). The cracking assay was initiated by incubating fruit in deionized water. At 2, 4, 6, 10 and 22 h after incubation, fruit were inspected for cracks. Fruit that cracked was discontinued. Non-cracked fruit was returned and—after refreshing the incubation medium—incubation was continued for the next time interval. The number and mass of fruit that cracked at any time was recorded. Fruit that cracked in the course of the experiment were frozen and held at -19°C for Ca analyses. The experiment was carried out at ambient temperature. From the number of fruit cracked at different time the cracking index (CI) was calculated according to:

$$CI=\frac{(5a+3b+c)\;*\;100}{250}$$

In this equation, a, b and c represent the number of fruit that cracked after 2, 4, and 6 h [38, 39]. A high CI means that a genotype cracks sooner than one with a low CI that cracks more slowly. The total number of fruit that cracked may not differ.

Typically, cracking increases in a sigmoidal pattern with time. Since cracking was also assessed at 10 and 22 h, it was possible to fit a four-parameter sigmoidal regression model to the time course of cracking [22]:

$$\mathrm{Cracking}\;(\%)=y_{0}+\frac{a}{1+{\text{e}}^{\frac{-\;\left(\text{time}\;(\text{h})-{\text{x}}_{0}\right)}{\text{b}}}}$$

In this equation a represents the asymptote, x_0_ the time at which cracking begins. The y_o_ is the amount of cracked fruit at time zero (typically 0) and b a scaling factor that affects the rate of cracking. When equating the percentage of cracking to 50% and solving for time, the half time (T_50_ in h) of fruit cracking is obtained [22]:

$${\text{T}}_{50}\;(\text{h})=-\ln\;\left(\frac{\text{a}}{50-{\text{y}}_{0}}-1\right)*\;\text{b}+{\text{x}}_{0}$$

This is the duration of incubation at which 50% of the fruit had cracked. With a single exception, cracking experiments were carried out using three replicates of 25 fruit each.

#### Cuticle mass per unit area

Epidermal skin segments (ES) were excised from the cheek of the fruit using a biopsy punch (8 mm diameter; Kai Europe, Solingen, Germany). The cheek was selected because of (1) the lower frequency of microcracks on the cheek and the requirement of intact cuticle discs for strain relaxation analysis and (2) the lower curvature of the cheek. The ESs consisted of the cuticular membrane (CM), an epidermis, hypodermal cell layers and adhering flesh. The CMs were enzymatically isolated by incubation in a solution of pectinase (90 ml l^−1^; Panzym Super E flüssig, Novozymes A/S, Krogshoejvej, Bagsvaerd, Denmark), and cellulase (5 ml l^−1^; Cellubrix L; Novozymes A/S) buffered in 50 mM citric acid buffer [40]. The pH was adjusted to pH 4.0 using NaOH. Sodium azide (NaN_3_) was added to prevent microbial growth. The final NaN_3_ concentration was 30 mM. Since the sweet cherry fruit skin is markedly strained [41], it is important to excise only one ES per fruit. After the CMs separated from adhering tissue, the CMs were carefully rinsed in deionized water and–when needed–cleaned with a soft aquarelle brush to remove any remaining cellular debris. The CMs were then dried on Teflon discs above dry silica gel and individually weighed on a microbalance. Cuticular wax was extracted by incubating individual CM discs for 48 h in 2.5 ml of chloroform/methanol (1/1 v/v). The so obtained dewaxed CM (DCM) were dried on Teflon discs above silica gel and then reweighed. The masses of the CM and DCM per unit fruit surface area were calculated. The amount of wax per unit area was obtained by subtracting the mass per unit area of the DCM from that of the CM. The number of replicates was 10 where an individual CM or DCM disc represented one replicate.

#### Biaxial strain relaxation

Since cuticle deposition ceases early during fruit development, the fruit cuticle of sweet cherry fruit becomes markedly strained [15, 42]. A portion of this strain is elastic and hence, reversible. This is released when the ES is excised and the CM is isolated and separated from adhering tissue [41].

The apparent strain release after excision and isolation of the CMs was quantified using the procedure described by [43]. Briefly, the cleaned CMs were spread on a microscope slide and flattened using a cover slip. Calibrated images were taken using a digitial camera (Camera DP71; Olympus; Tokio, Japan) and a binocular microscope (MZ10F; Leica Microsystems, Wetzlar, Germany). The area of the flattened disc after isolation (A_CM_) was measured by image analysis (cellSens Dimension 1.18; Olympus Soft Imaging Solutions, Münster, Germany). The strain release was calculated from the following equation [43]:

$$\text{Stain}\;\text{release}\;(\%)=\frac{A_{\text{i}}-A_{CM}}{{\text{A}}_{CM}}\times 100$$

In this equation A_i_ represents the area of the disc on the fruit before excision, i.e., the cross-sectional area of the biopsy punch, and A_CM_ the area of the isolated relaxed CM. The number of individual CM replicates ranged from 8 to 19.

#### Microcracking of the cuticular membrane

Microcracking of the strained cuticle is an early event in cracking of sweet cherry fruit and the stylar end region of the fruit is most susceptible to microcracking [17]. Microcracking was quantified before and after a 2 h incubation period in deionized water. Microcracking was indexed by the area infiltrated with the fluorescent tracer acridine orange. Acridine orange does not penetrate an intact cuticle and therefore, penetration is restricted to microcracks in the cuticle. Briefly, fruit were placed in the wells of a 6-well multiwell-plate. The wells were filled with an 0.1% aqueous acridine orange solution. The fruit was positioned such that the stylar end region was in contact with the acridine orange solution. After 10 min, fruit were removed from solution, blotted, rinsed with deionized water and then viewed under a binocular microscope (Axio Zoom.V16; Zeiss, Jena, Germany). Settings were: objective 0.5x, magnification 6.5x, 72% aperture, exposure time 120 ms. One digital calibrated image per fruit of the stylar end region was taken under white light and fluorescent light using the GFP Filter (excitation wave length 460–488 nm, emission wave length > 496 nm). The size of the microscope window was 17.4 mm × 13.0 mm equivalent to an area of 226.6 mm^2^. Subsequently, microcracks were induced by incubating fruit for 2 h in deionized water. Fruit were blotted and re-photographed under fluorescent light as described above. Microcracking of the cuticle was indexed by quantifying the fluorescing area around the stylar scar of the fruit before and after the 2 h induction period in deionized water using image analysis (cellSens Dimension 1.18; Olympus Soft Imaging Solutions, Münster, Germany). The increase in infiltrated area (‘Δ infiltrated area’) was calculated by subtracting the area infiltrated after the 2 h incubation period from that before. The area of the stylar scar was measured separately and subtracted and hence, is not included in the analysis.

#### Ca analysis

The fruit that cracked in the course of the cracking assays were pooled from the three replicates of the cracking assay, weighed and their Ca content quantified such that fruit that cracked at different times could be compared. The Ca analysis was conducted as described in detail by [44]. Briefly, frozen fruit from the cracking experiment were cut perpendicular to the longitudinal axis above and below the pit into a proximal stem end section, an equatorial section and a distal stylar end section. The pit was removed from the equatorial section and discarded. The three sections were then weighed, crushed, freeze dried, oven dried at 103°C to constant mass, re-weighed for dry mass determination and then ground (MM 400; Retsch, Haan, Germany). An aliquot of each section was taken and re-dried to constant mass at 103°C. About 100 mg of the dried powder of each section was ashed at 500°C in a muffle furnace (L24/11/B180; Nabertherm, Lilienthal, Germany). The ash was taken up in 2 ml of 1 M HCl and 8 ml deionized water and filtered (MN 640 m; Macherey-Nagel, Düren, Germany). The filtrate was diluted with deionized water as needed. The solutions were analyzed using atomic absorption spectrometry (AAS) (AAS 1100B; Perkin Elmer, Waltham, MA, USA) equipped with a Ca lumina hollow cathode lamp (wavelength 422.7 nm, slit 0.7 nm) using an air-acetylene flame. Lanthanum chloride (LaCl_3_) was added to the solution at a concentration of 1% to eliminate interference between Ca and phosphorus [45]. Earlier studies revealed that without LaCl_3_ the Ca content of sweet cherry is underestimated by about 50% [46]. Since the amount of Ca per fruit is positively related to fruit mass, the fruit Ca content was expressed as the Ca/dry mass ratio (mg g^-1^ dry mass). The analysis was carried out with three technical replicates.

### Data analysis

Data are presented in figures and tables as means ± SE. Where not shown, SE bars were smaller than data symbols. The only exception were the Ca data where fruit samples from the three replicates of the cracking assays had to be pooled due to the limiting fruit mass particularly at the later time points. Here, only two to three technical replicates were analyzed. Correlation and regression analyses and analyses of variance were carried out using the statistical software package SAS (version 9.4; SAS Institute, Cary, NC, USA).

## Results

**Fig 1:**
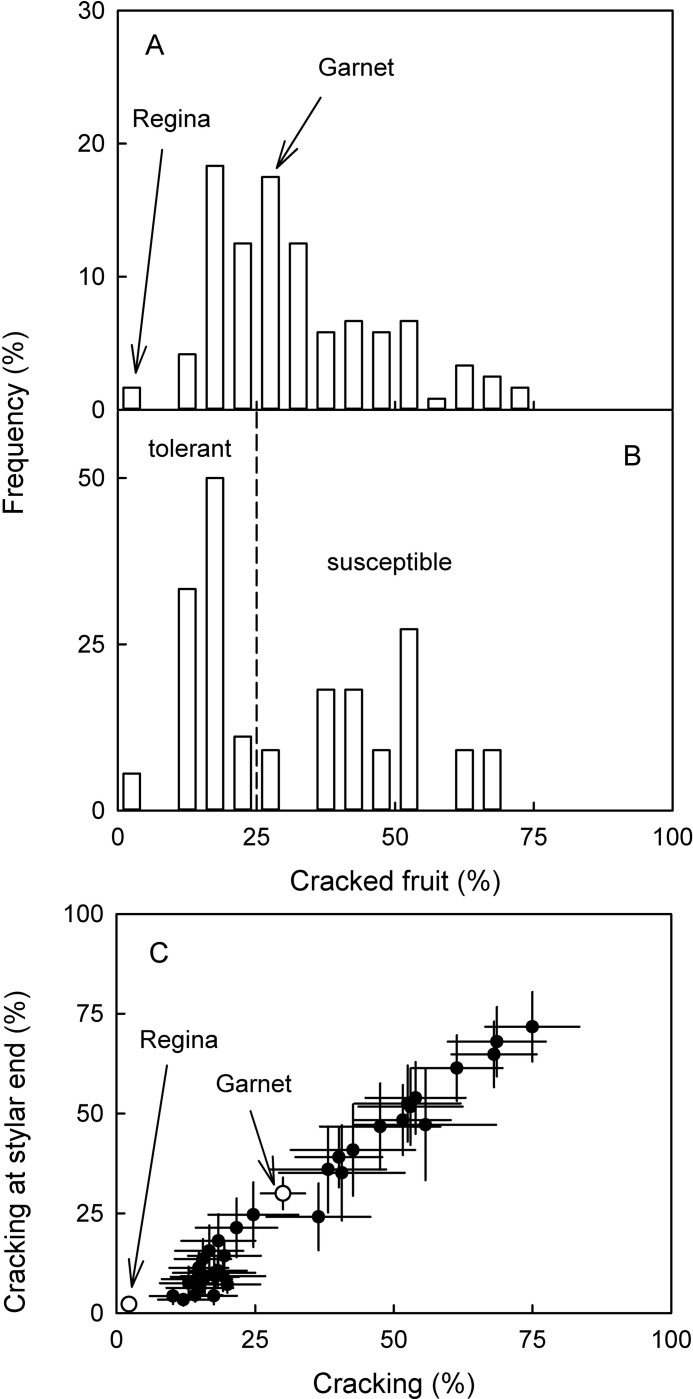
Frequency distribution of cracking susceptibility of sweet cherry genotypes. (A) Segregating population of full siblings of a ‘Regina’ x ‘Garnet’ cross comprising 117 individuals. The percentage of fruit cracking is based on 8 years of cracking assessment in the field following exposure to natural rain. (B) Partitioning of genotypes from A that had sufficient fruit set in the 2022 growing season into two categories of contrasting cracking susceptibility. Vertical dashed line represents the arbitrary threshold of 25% cracking that was chosen to separate the population into cracking tolerant and cracking susceptible genotypes. (C) Relationship between frequencies of stylar end cracking and overall fruit cracking irrespective of crack position as observed in eight growing seasons following exposure to natural rain. Open symbols in C represent the ‘Regina’ and ‘Garnet’ parent.

The total population of 117 siblings of the ‘Regina’ × ‘Garnet’ cross varied widely in their cracking susceptibility based on eight years data of cracking in the field (Fig 1A). The siblings covered a range in cracking susceptibility that was larger than the difference between the two parents. None of the siblings was more tolerant than ‘Regina’, but 15 siblings were more susceptible than ‘Garnet’.

After evaluating final fruit set and June drop in the 2022 growing season, a subpopulation of 29 genotypes was selected that was partitioned into a category of cracking tolerant and cracking susceptible individuals using an arbitrary threshold of 25% cracking in the field (averaged over eight years) (Fig 1B). Subsequent statistical analysis revealed that these two categories differed significantly in the percentage of maximum fruit cracking, mean fruit cracking, predicted cracking, the percentage of fruit with stylar end cracking, equatorial cracking and stem end cracking (Table 1). Stylar end cracking and maximum cracking across regions were closely related (r = 0.98***) indicating that cracking occurred most frequently in the stylar scar region (Fig 1C). There was no significant difference in fruit mass between tolerant and susceptible genotypes. Also, there was no significant correlation between cracking susceptibility and fruit mass across all genotypes.

**Table 1:** Compilation of crack characteristics of sweet cherry fruit from a segregating population of a cross of ‘Regina’ x ‘Garnet’. Cracking was assessed in a laboratory based immersion assay (n = 3) using the cracking index [38, 39], the time to half maximum cracking (T_50_) or the percentage of fruit cracked at infinity (asymptote) using fruit from grafted trees. Cracking in the field was recorded in eight growing seasons following exposure to natural rain using the same genotypes as above but on their own roots. These data are taken from Quero-Garcia, Letourmy [33]. The number of replicates was 16 susceptible and 21 tolerant genotypes including the parent cultivars Regina and Garnet.

Characteristic	Unit	Genotype (means ± SE)		
		Tolerant	Susceptible	P-Value
Lab assay (grafted trees)				
Cracking index	(%)	52.8 ± 4.4	77.7 ± 3.4	0.0002
T_50_	(h)	3.7 ± 0.4	2.1 ± 0.1	0.0015
Asymptote	(%)	93.5 ± 1.0	96.7 ± 0.7	0.0202
Field ratings (on their own roots)				
Stylar end cracking	(%)	10.3 ± 1.3	48.2 ± 3.4	< .0001
Cheek/suture cracking	(%)	8.9 ± 0.9	20.6 ± 2.5	< .0001
Stem cavity cracking	(%)	9.7 ± 1.2	24.8 ± 2.2	< .0001
Mean cracking across regions	(%)	9.6 ± 0.6	31.2 ± 1.8	< .0001
Maximum cracking across regions	(%)	16.1 ± 1.0	50.9 ± 3.2	< .0001
Predicted cracking	(%)	26.7 + 2.0	73.4 + 3.9	< .0001

All comparisons within rows significantly different according to Tukey’ Studentized range test.

Mean cracking and maximum cracking represent the mean and the maximum of stylar end, cheek/suture and stem end cracking per genotype per year. The predicted cracking represents a cracking percentage that is corrected for fruit mass, firmness and amount of rain received per genotype per year. For details see [33].

**Fig 2:**
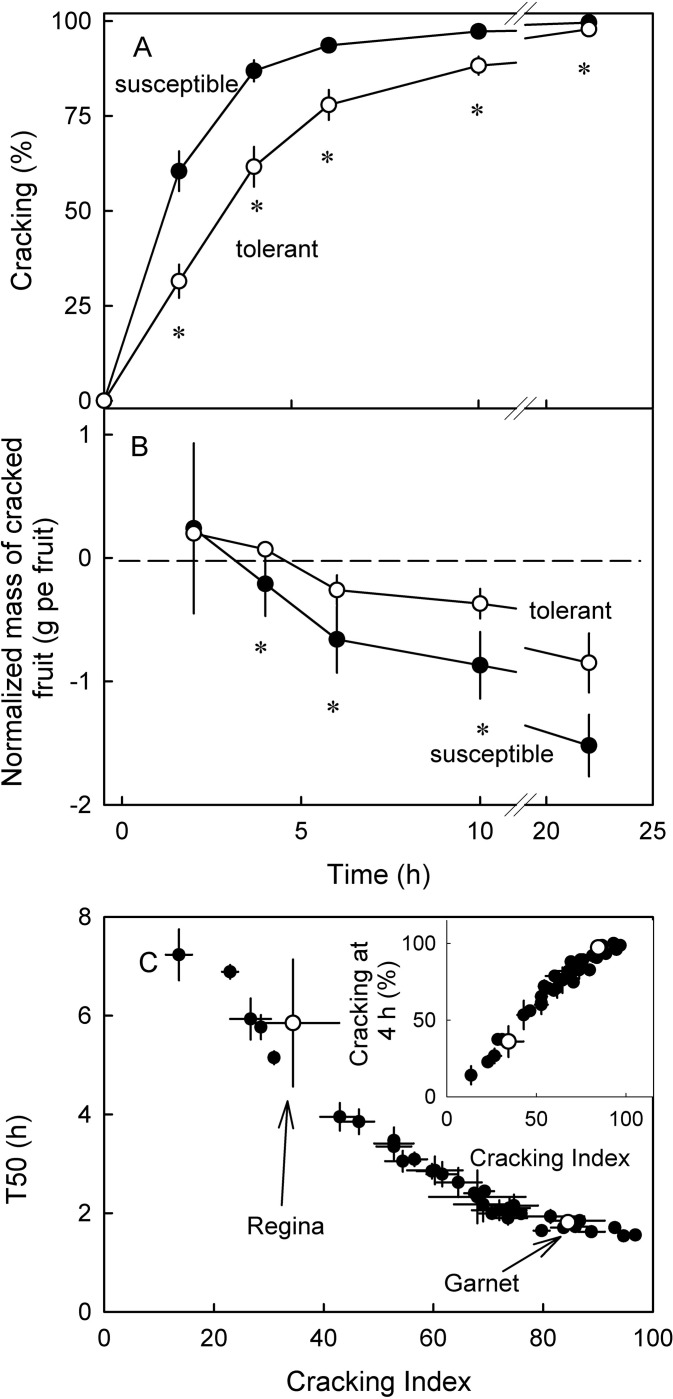
(A) Time course of cracking of susceptible and tolerant genotypes when using an immersion assay in deionized water. (B) Normalized fruit mass in the course of an immersion assay. Dashed horizontal line indicates the mean fruit mass of all fruit in the experiment. Asterisk indicate significant differences, Tukey’s Studentized range test, P = 0.05. (C) Relationship between the time to half maximum cracking (T_50_) and the cracking index as described by [38, 39]. Inset: Relationship between the percentage of fruit cracking at 4 h and the cracking index. Open symbols in C represent the ‘Regina’ and ‘Garnet’ parent.

When subjecting these genotypes to a laboratory-based immersion assay, cracking increased in a sigmoidal pattern with time. The increase was more rapid for susceptible than for tolerant genotypes. Fruit from susceptible genotypes cracked significantly more frequent at all times than those of tolerant genotypes (Fig 2A). The cracking index, the T50 and the amount of cracking at infinity all differed significantly between the susceptible and tolerant genotypes (Table 1). Within both categories of susceptibility, the larger fruit were more susceptible to cracking than the smaller fruit (Fig 2B). Furthermore, as incubation time progressed, the fruit from susceptible genotypes that cracked were smaller and smaller than those of tolerant ones. Across all genotypes the T50 and the cracking index or the percentage cracking at 4 h and the cracking index were closely related (Fig 2C).

Cracking assessments in the field were significantly related to cracking in the immersion assay for stylar end cracking, stem end cracking, mean and maximum cracking, but not for cracking in the equatorial region, i.e., cracking on the cheek or suture (Table 2). The coefficients of correlation were highest for the relationship with the cracking index. As incubation time progressed, coefficients of correlation between cracking in the field and in the immersion assay consistently decreased and at 22 h of immersion became non-significant. Interestingly, the percentages of cracking after 2 h or 4 h of incubation were nearly as closely related to maximum cracking in the field as was the cracking index or the T_50_ that required three (2,4 and 6 h for the CI) or even five determinations of cracking (at 2,4,6,10 and 22h for the T_50_), respectively (Table 2).

**Table 2:** Coefficients of correlation between cracking as quantified in the lab using an immersion assay and fruit from grafted trees and as observed in the field using trees on their own roots that were exposed to natural rain. Fruit cracking in the lab was quantified using the cracking index (‘CI’), the time to half maximum cracking (‘T_50_’) and the percentage of fruit that cracked at infinity (‘asymptote’). Cracking in the field was assessed as the percentage of fruit that cracked in the proximal stem cavity, the equatorial region of cheek and suture and the distal stylar end cracking, the mean cracking, the maximum and the predicted cracking percentage. For details see text.

Immersion assay in the lab	Pearson coefficient of correlation (r)					
	Cracking in the field in eight years					
	Stem cavity	Equatorial region	Stylar end region	Mean cracking	Maximum cracking	Predicted cracking
Cracking at 2 h	0.45**	0.23n.s.	0.52***	0.50**	0.53***	0.52**
Cracking at 4 h	0.41*	0.15ns	0.52**	0.47**	0.52**	0.51**
Cracking at 6 h	0.35*	0.11ns	0.47**	0.41*	0.46**	0.45**
Cracking at 10 h	0.38*	0.16ns	0.46**	0.43**	0.47**	0.42*
Cracking at 22 h	0.28ns	0.06ns	0.35*	0.31ns	0.36*	0.32ns
Cracking index	0.44**	0.19n.s.	0.53***	0.50**	0.54***	0.53**
T50	-0.49**	-0.20ns	-0.49**	-0.46**	-0.49**	-0.47**S
Asymptote	0.32ns	-0.02ns	0.23ns	0.25ns	0.32ns	0.31ns

Mean cracking and maximum cracking represent the mean and the maximum of stylar end, cheek/suture and stem end cracking per genotype per year. The predicted cracking represents a cracking percentage that is corrected for fruit mass, firmness and amount of rain received per genotype per year. For details see [33]. The number of replicates was 37 genotypes including the parent cultivars Regina and Garnet. The only exception was for predicted cracking, where data for parent cultivars were not available.

Significance of the coefficient of correlation is indicated by *, ** and *** at P = 0.05, 0.01 and 0.001. ns, not significant.

**Fig 3:**
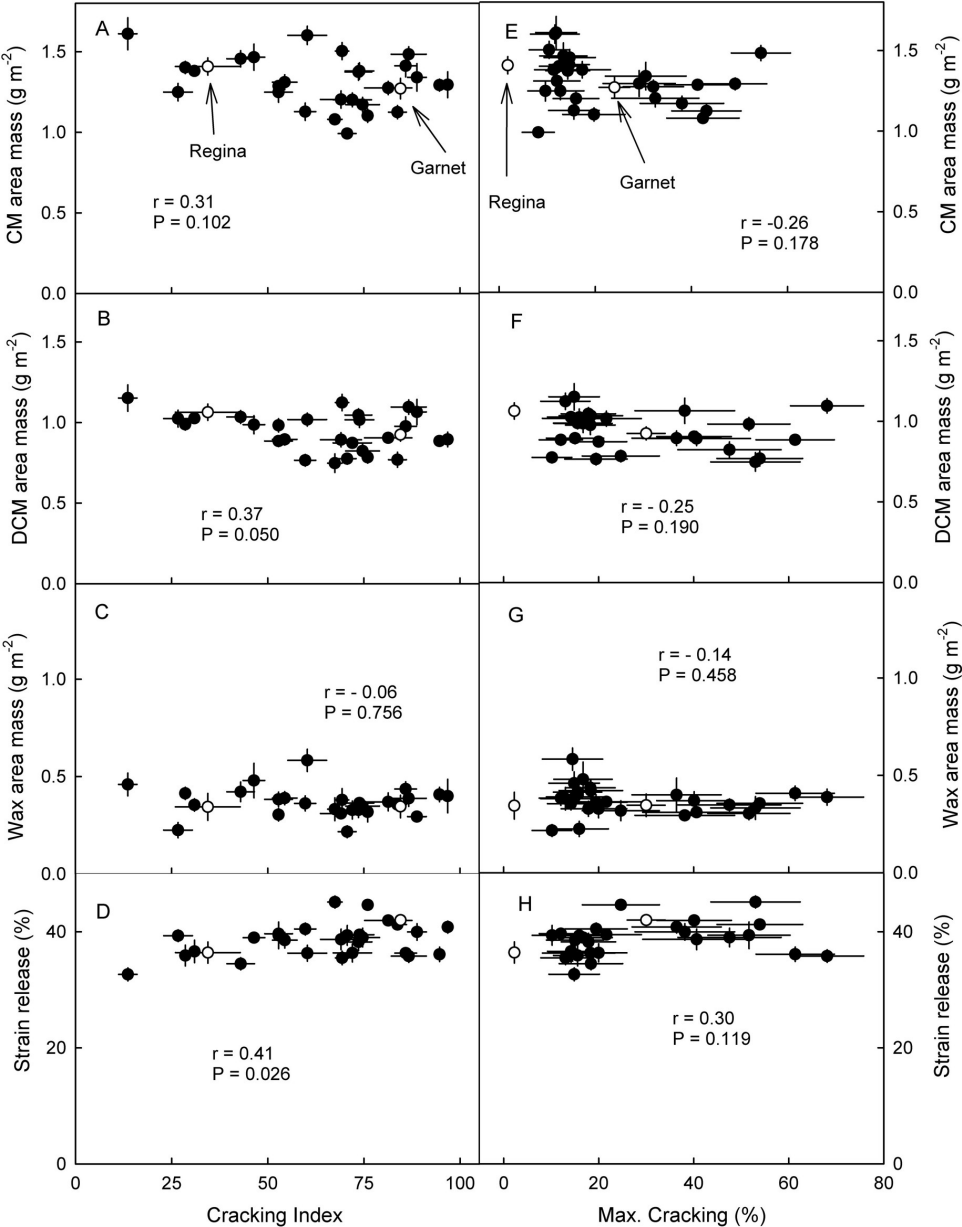
Relationship between cuticle properties of mature sweet cherry fruit of siblings of a cross of ‘Regina’ x ‘Garnet’ and their cracking susceptibility as assessed in a laboratory based incubation assay as indexed by the cracking index (A,B,C,D) or as observed in eight growing seasons following exposure to natural rain (E,F,G,H). A,E. Mass per unit fruit surface area of the cuticular membrane (CM). B,F. Mass per unit area of the dewaxed CM (DCM). C,G. Mass per unit area of cuticular wax. D,H. Release of strain of the CM following excision and isolation. Open symbols represent the ‘Regina’ and ‘Garnet’ parent.

**Fig 4:**
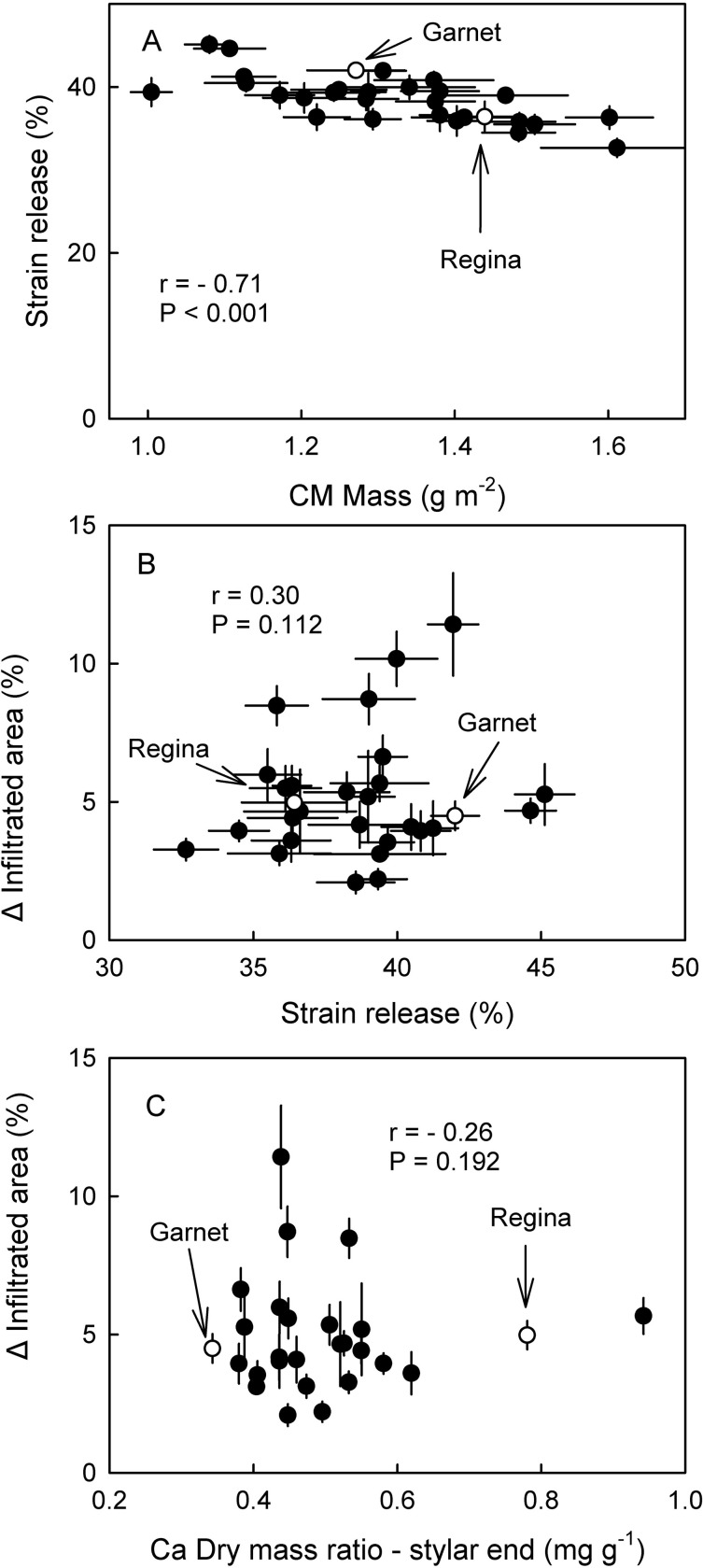
(A). Strain release of the cuticular membrane (CM) as affected by the mass of the CM per unit surface area of fruit of mature sweet cherry siblings of a cross of ‘Regina’ x ‘Garnet’. (B). Relationship between the extent of microcracking of the cuticle and the amount of strain relaxation following excision and isolation of the cuticular membrane. Microcracking was indexed in the stylar end region by the increase in area infiltrated with the fluorescent tracer acridine orange during a 2 h incubation period in deionized water. (C). Relationship between the extent of microcracking of the cuticle and Ca/dry mass ratio in the stylar end region of sweet cherry fruit. Open symbols represent the ‘Regina’ and ‘Garnet’ parent.

There was no significant relationship between the masses of the cuticular membrane (CM) per unit area or that of the wax per unit area and the cracking index (Fig 3A–3D). Only for the DCM mass per unit area and the strain relaxation of the cuticle was this relationship significant. All correlations between cuticle mass or strain release and fruit cracking in the field were not significant (Fig 3E–3H). The strain released from the cuticle after isolation was significantly and negatively correlated with the cuticle mass per unit fruit surface area (Fig 4A) indicating that thicker cuticles were less strained.

**Fig 5:**
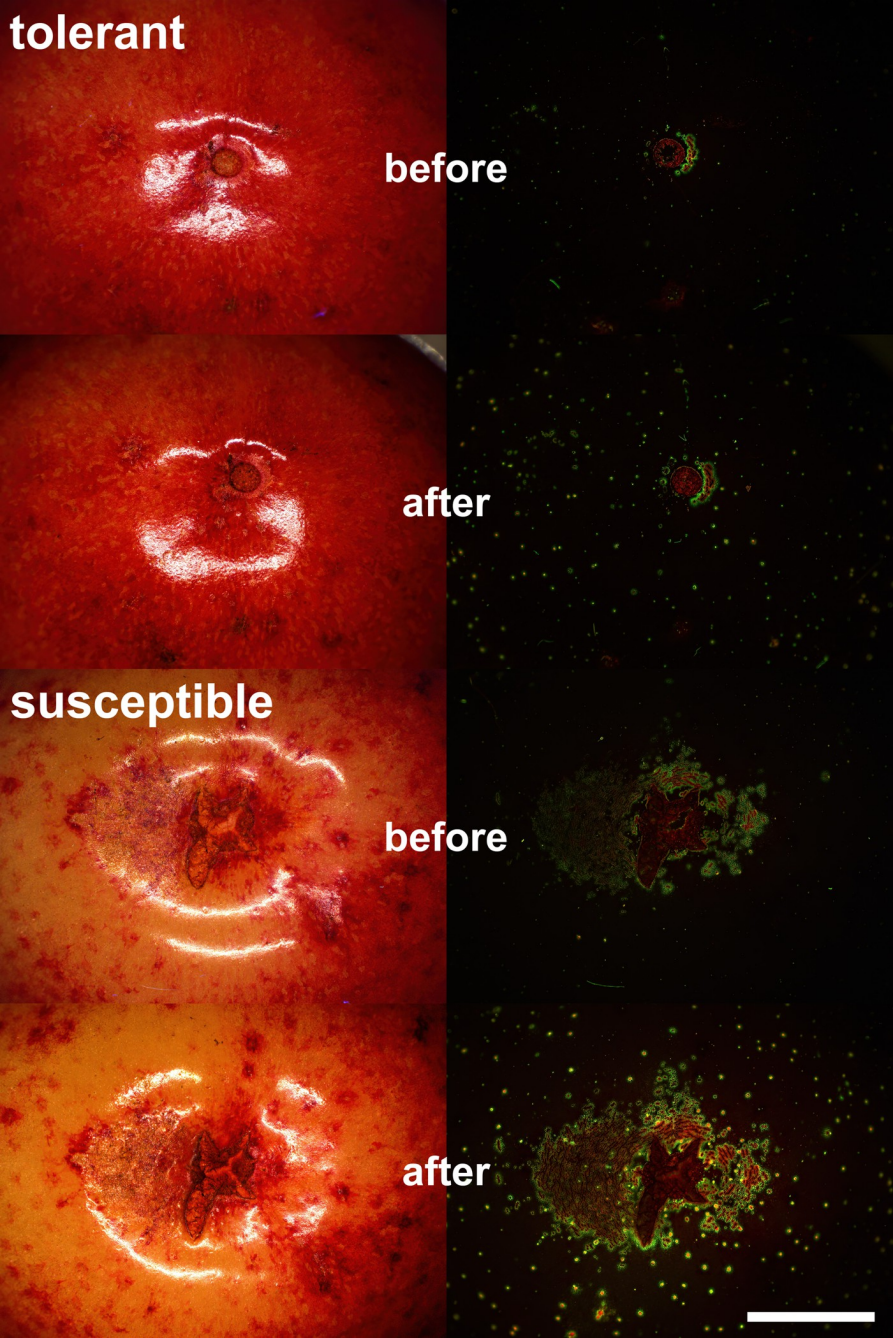
Representative micrographs of the stylar end region of fruit of a cracking susceptible (RxG113) and cracking tolerant genotype (RxG80) before and after a 2 h incubation period in deionized water for induction of microscopic cracks (‘microcracks’) in the cuticle. Fruit were viewed in incident white light or fluorescent light. Microcracks were stained for 10 min using the fluorescent tracer acridine orange. Penetration of acridine orange is limited to microcracks in the cuticle and does not occur through an intact fruit cuticle. Scale bar for all micrographs = 5 mm.

**Fig 6:**
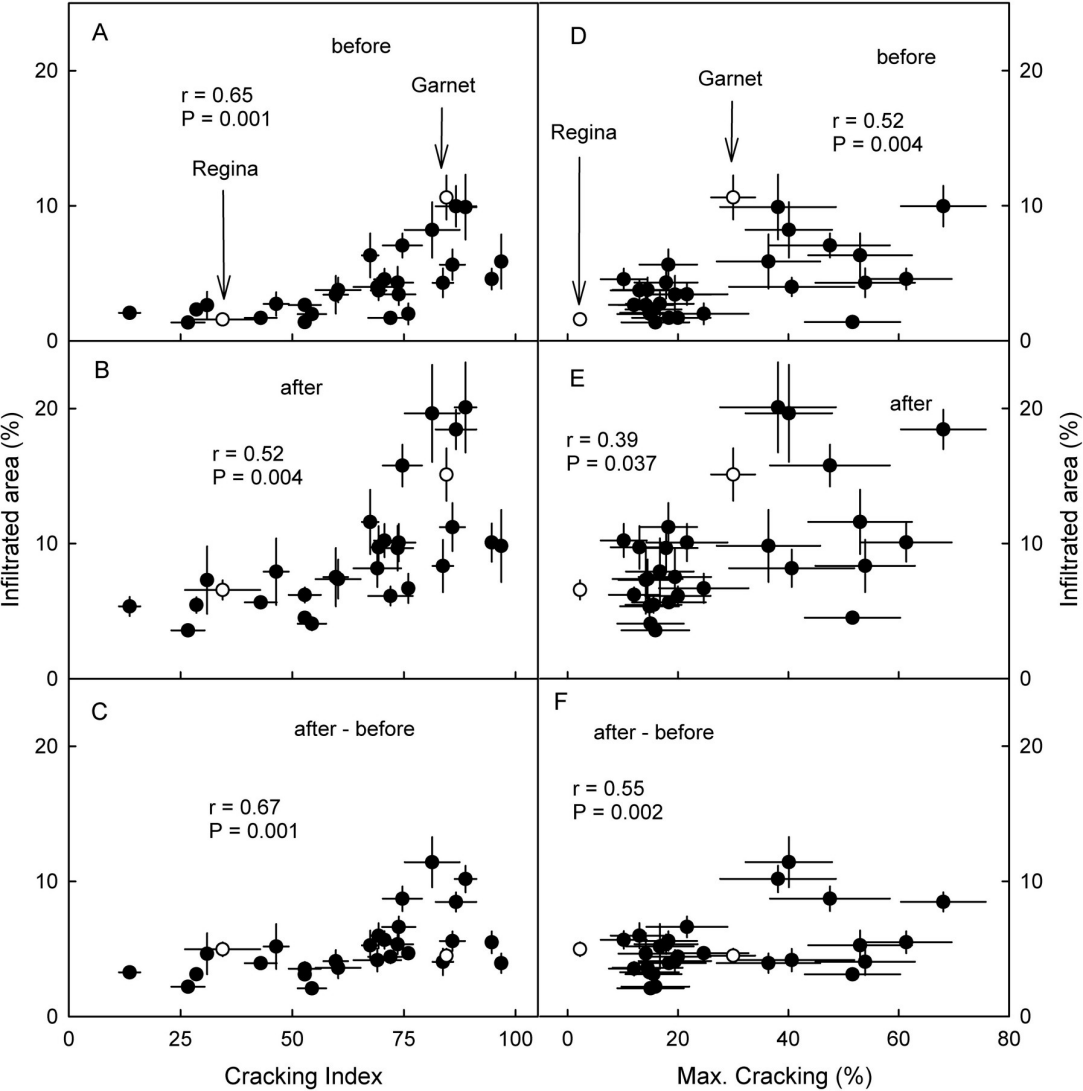
Relationship between microcracking of the cuticle in the stylar end region of mature fruit of siblings of a cross of ‘Regina’ x ‘Garnet’ sweet cherry and their cracking susceptibility as assessed in a laboratory based incubation assay as indexed by the cracking index (A,B,C) or as observed in eight growing seasons following exposure to natural rain (D,E,F). Microcracking was indexed by the area infiltrated with the fluorescent tracer acridine orange. Fruit were inspected before and after a 2 h incubation period in deionized water for microcrack induction and the area infiltrated by acridine orange in the stylar end region of the fruit quantified using fluorescence microscopy and image analysis. A,D. Infiltrated area before induction of microcracks. B,E. Infiltrated area after induction of microcracks. C,F. Increase infiltrated area after induction of microcracks. The increase in infiltrated area (Δ Infiltrated) was calculated as the difference between the infiltrated area after minus that before incubation. Open symbols represent the ‘Regina’ and ‘Garnet’ parent.

Microcracking of cuticle in the stylar end region as indexed by the area infiltrated with the fluorescent tracer acridine orange was more severe in susceptible than in tolerant genotypes (Fig 5). Most microcracks were tangentially oriented relative to the stylar scar and often slightly extending into the suture (Fig 5). Correlation analysis revealed significant relationships between the areas infiltrated before and after the 2 h induction period of microcracks and the increase in microcracking during the induction period and both, laboratory and field assessments of cracking susceptibilities (Fig 6). Coefficients of correlation were significantly higher for laboratory than for field assessments. It is worth noting that the increase in microcracking during the 2 h induction period was very closely related to the amount of microcracking before the induction (r = 0.94***) indicating that it didn’t matter whether the microcracking resulted from natural non-standardized rain or the standardized exposure of rain.

Surprisingly, the relationship between the increase in microcracking and the strain release of the cuticle on isolation was not significant (Fig 4B).

**Fig 7:**
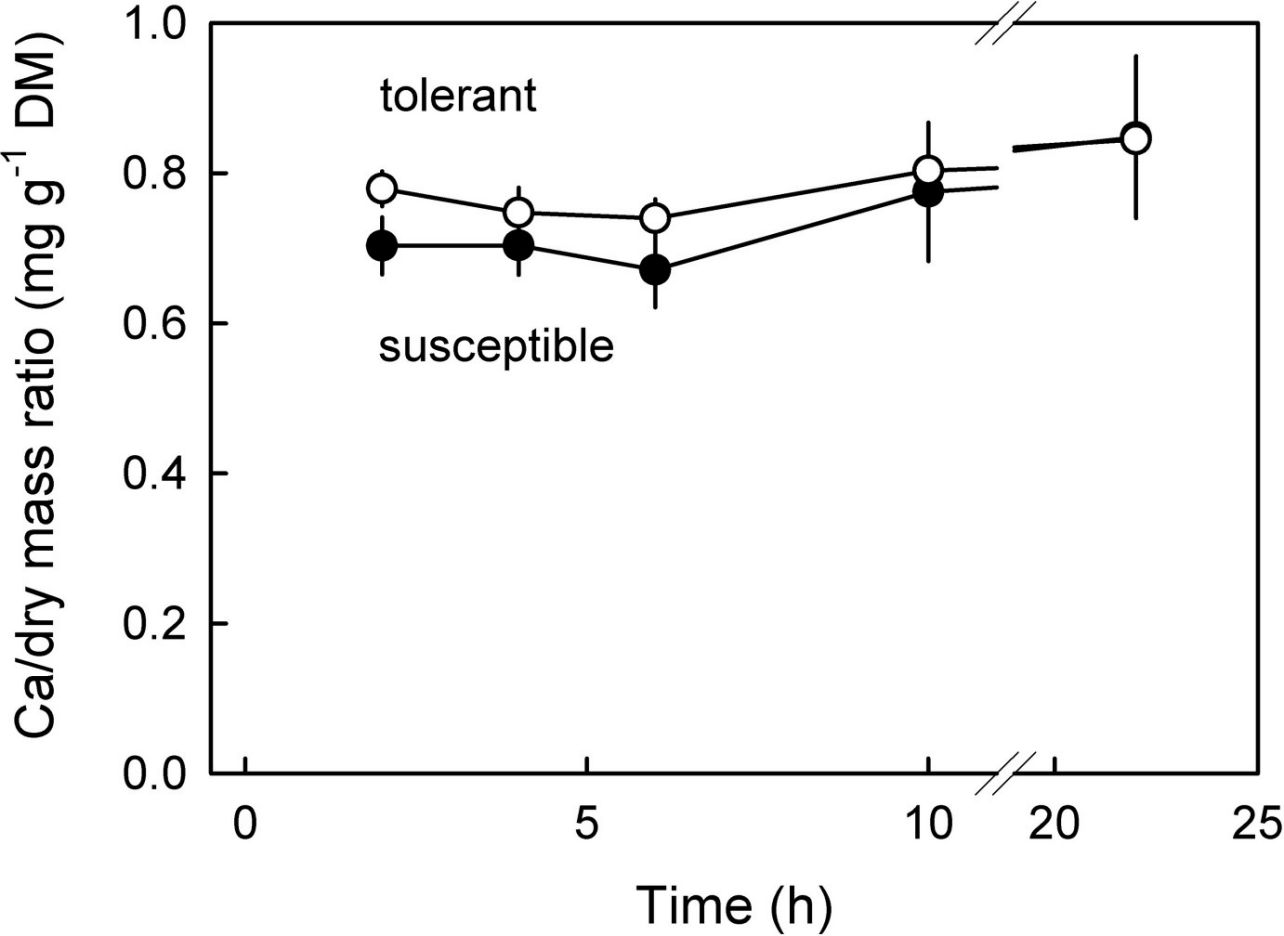
Time course of change in Ca/dry mass ratio of fruit of cracking tolerant and cracking susceptible sweet cherry genotypes that cracked in the course of a laboratory-based incubation assay for quantifying cracking susceptibility.

The average Ca/dry mass ratio of tolerant genotypes exceeded that of susceptible genotypes by about 8% (Table 3). For both categories of susceptibility, a steep gradient in Ca/dry mass ratio along the stem/stylar scar axis was observed. The Ca/dry mass ratio markedly decreased from the stem end to the equatorial region to the distal stylar scar region. It is interesting to note that the Ca/dry mass ratio of fruit that cracked at 10 or 22 h of incubation was significantly higher than that of fruit that cracked at 2,4 and 6 h (Fig 7).

**Table 3:** Ca/dry mass ratio in the proximal stem end, the equatorial and the distal stylar end region of fruit of cracking susceptible and tolerant genotypes. Fruit were taken from a segregating population of grafted trees of siblings of a cross of ‘Regina’ × ‘Garnet’.

Cracking	Ca/dry mass ration (mg g^-1^ DM)			
	Stem end	Equatorial	Stylar end	Mean
Susceptible	1.21	0.63	0.47	0.71 b
Tolerant	1.29	0.65	0.51	0.76 a
Mean	1.25 a	0.64 b	0.49 c	

Two factorial analysis of variance using time as a covariable revealed significant main effects for category of cracking susceptibility and region per fruit. The interaction was not significant. Mean separation within main effects by Tukey’s studentized range test, P = 0.05. The number of replicates ranged from 56 to 99.

**Fig 8:**
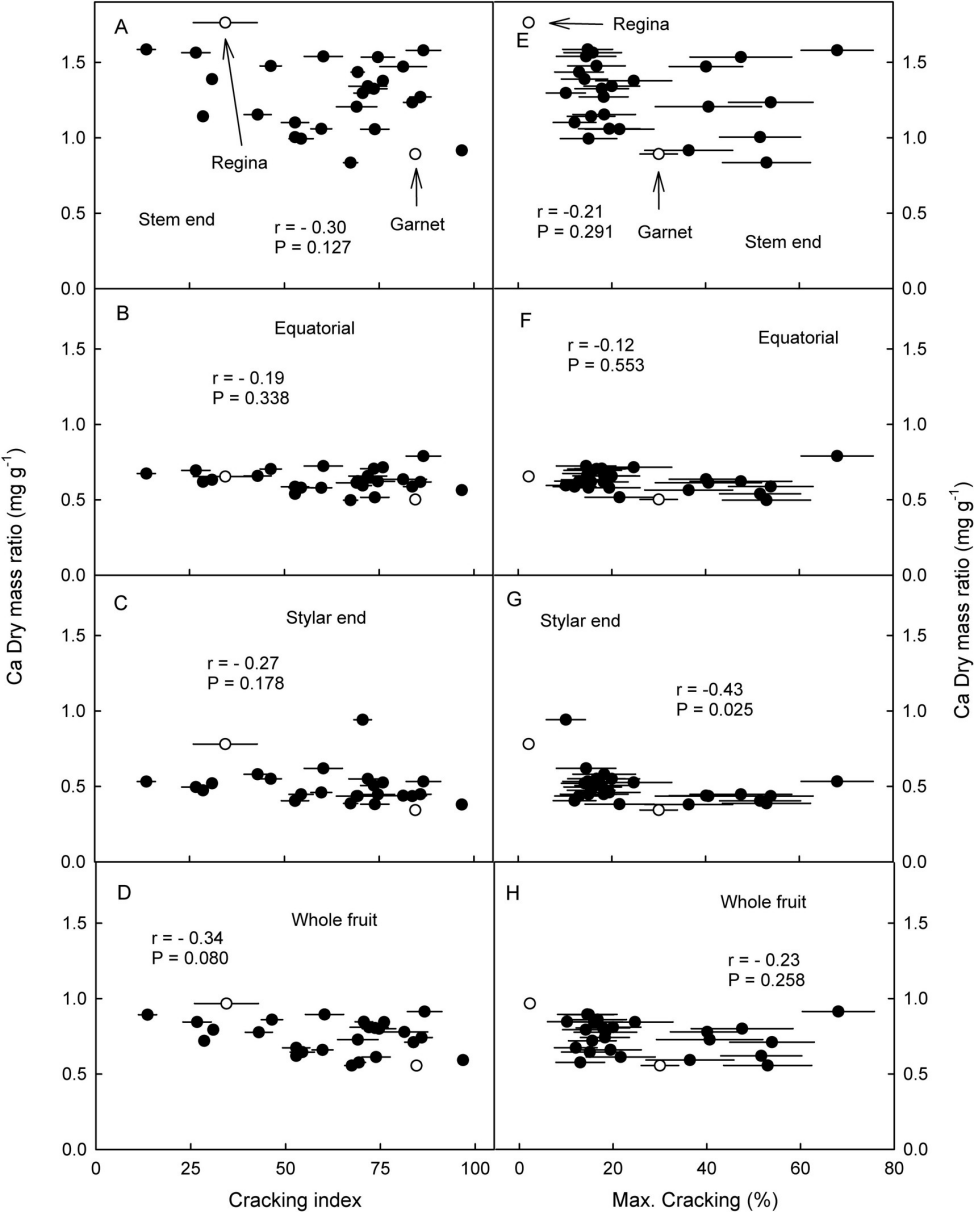
Relationship between the Ca/dry mass ratio of mature fruit of siblings of a cross of Regina x Garnet and their cracking susceptibility as assessed in a laboratory based incubation assay as indexed by the cracking index (A,B,C,D) or as observed in eight growing seasons following exposure to natural rain (E,F,G,H). The Ca/dry mass ratio was determined in the stem end region (A,E), the equatorial region (B,F) the stylar end region (C,G) and as a the average Ca/dry mass ratio per fruit without pit (D,H). Open symbols represent the ‘Regina’ and ‘Garnet’ parent.

Relationships between the Ca/dry mass ratio in stem end, equatorial and stylar end regions of the fruit and the cracking susceptibility as assessed by the cracking index in the lab or by field assessments were not significant (Fig 8). The only exception was a significant negative correlation between the Ca/dry mass ratio in the stylar end region and the field assessments of cracking where fruit having a higher Ca/dry mass ratio was less susceptible to cracking. There was no relationship between the Ca/dry mass ratio in the stylar end region and the extent of microcracking (Fig 4C).

Subjecting the whole data set to a stepwise multiple linear regression analysis identified the increase in microcracking during a 2 h incubation period in acridine orange as the variable that explained most of the cracking susceptibility. This finding was consistent for both, eight years of cracking observations on seedlings trees in the field (partial r^2^ = 0.331 to 0.384; Table 4) and the incubation assay using fruit from grafted trees in the lab (partial r^2^ = 0.326 to 0.453; Table 4). The second variable entered into the regression model differed between the laboratory assessment of cracking susceptibility and the field observations. For the laboratory assessment the mass of the DCM per unit area was selected, whereas for the field data it was the Ca/dry mass ratio in the stylar end region (percentage of maximum cracking or stylar end cracking) or the strain release of the cuticle on isolation (predicted percentage of cracking). Of the total variability in cracking susceptibility in the lab up to 57% was accounted for by microcracking and DCM mass, for that in the field the variability accounted for was up to 48% (Table 4).

**Table 4:** Parameters of multiple linear regression equations explaining sweet cherry fruit cracking in a laboratory based immersion assay or as observed in eight years in the field following exposure to natural rain. Cracking in the immersion assay was indexed by the cracking index or the time to half maximum cracking (T_50_). In the field the percentage of fruit that cracked or fruit that cracked in the stylar end region was recorded [33]. The same genotypes were used for laboratory and field assessments. Fruit for the laboratory assay was from grafted trees, the field observations were done on trees of the same genotpyes on their own roots.

Site	Dependent Variable	n	Parameters	Estimate ± SE	Partial r^2^	r^2^	P>F
Lab	Cracking index	27	Intercept	100.1 ± 24.4			0.0004
			Δ Infiltrated area (%)	5.3 ± 1.1	0.453	0.453	0.0001
			DCM (g m^-2^)	-63.7 ± 24.8	0.118	0.571	0.0167
	T_50_ (h)	27	Intercept	-1.01 ± 1.94			0.6066
			Δ Infiltrated area (%)	-0.32 ± 0.09	0.326	0.326	0.0016
			DCM (g m^-2^)	5.75 ± 1.96	0.177	0.503	0.0074
Field	Maximum crack (%)	27	Intercept	32.8 ± 12.7			0.0161
			Δ Infiltrated area (%)	3.3 ± 1.0	0.336	0.336	0.0047
			Ca/dry mass ratio in stylar end (mg g^-1^)	-39.9 ± 21.2	0.085	0.421	0.0728
	Stylar end cracking (%)	27	Intercept	26.1 ± 14.1			0.0764
			Δ Infiltrated area (%)	3.6 ± 1.2	0.331	0.331	0.0051
			Ca/dry mass ratio in stylar end (mg g^-1^)	-38.0 ± 23.6	0.065	0.396	0.1204
	Predicted cracking (%)	25	Intercept	-77.9 ± 47.7			0.1168
			Δ Infiltrated area (%)	5.9 ± 1.6	0.384	0.384	0.0010
			Strain release (%)	2.5 ± 1.3	0.095	0.478	0.0582

Fruit were sampled from 27 genotypes including the ‘Regina’ x ‘Garnet’ parents. The only exception was the percentage of predicted cracking that was not available for the ‘Regina’ and ‘Garnet’ parents (n = 25).

Mean cracking and maximum cracking represent the mean and the maximum of stylar end, cheek/suture and stem end cracking per genotype per year. The predicted cracking represents a cracking percentage that is corrected for fruit mass and occurrence of rain per genotype per year. For details see [33]. The stepwise regression analysis was conducted using the SAS procedure Proc Stepwise. The default setting for entry level of variables into the model of 0.1500 was used. The independent variables included in the analyses were the masses per unit surface area of the cuticular membrane (CM), of the dewaxed CM (DCM) and of the wax, the infiltrated area in the stylar end region before and after induction of microcracks and the increase in infiltrated area during microcrack induction, the Ca/dry mass ratio in the proximal stem end, the equatorial and the distal stylar end regions and the mean fruit Ca/dry mass ratio.

## Discussion

In our discussion we focus on the following topics:

1. The relationship between assessments of cracking of fruit of seedling trees in the field with a laboratory assessment of fruit from grafted trees,
2. the relationships between cracking susceptibility and microcracking of the cuticle, DCM mass per unit area and the Ca/dry mass ratio in the stylar end
3. potential adaptations of the phenotyping protocol for screening of larger number of genotypes.

### Relationship between assessments of cracking of fruit from seedling trees in the field and a laboratory-based assessment of fruit from grafted trees

The unique dataset in this study allowed field observations on cracking of fruit from seedling trees to be related to a laboratory assessment of fruit from grafted trees of the same genotypes. The coefficients of correlation were significant suggesting that water on the skin was the primary factor in cracking. This excludes root uptake and movement via the pedicel as a major factor contributing to cracking under the temperate weather conditions of the experiment. It also implies that the scion genotype rather than the rootstock was the important factor. This is consistent with the essentially complete loss of xylem functionality in sweet cherry during development [47–49]. Also, based on the Zipper model, sweet cherry fruit cracking is a localized event that is initiated by the bursting of individual cells in the outer flesh [18, 20, 21, 50]. That coefficients of correlation between cracking susceptibilities in field and lab were fairly low may be attributed to one or several of the following: First, cracking observations in the field were not done in the same season as the physiological traits were investigated. Second, in the field amount and distribution of rainfall and dew varies between seasons. In addition, the genotypes differ in their rate of development and hence, in exposure. Both factors together cause considerable variability due to environmental factors. We expect these factors to be particularly important because they will affect fruit surface wetness duration. The surface wetness duration–in turn–is a critical factor in microcracking [1, 17]. These arguments also explain the consistently higher r when (laboratory) assessments of cracking are conducted in the same season that the extent of microcracking is quantified. In their initial work, [33] integrated environmental variability between seasons into linear models by considering as covariates the amount of rainfall that the fruit of each genotype had received before harvest, the mean fruit weight and firmness of each genotype. Unfortunately, there was no data on surface wetness duration which is more critical than the absolute amount of rainfall.

Our data also demonstrate that grading of fruit for representative size is essential for obtaining reproducible and consistent results between replicates. The first fruit to crack for anyone genotype always had an above average mass and the last fruit to crack was always below average mass. This was consistent for cracking tolerant and cracking susceptible genotypes.

### Relationships between cracking susceptibility and microcracking of the cuticle, DCM mass per unit area and the Ca/dry mass ratio in the stylar end

Cracking susceptibility was significantly related to microcracking of the cuticle, the DCM mass per unit area (lab incubation assay only), the Ca/dry mass ratio (percentages of stylar end cracking and maximum cracking, field data) and the extent of strain release (predicted percentage of cracking, field data). This finding is consistent with the Zipper model. Of these traits microcracking was the most important. It accounted for between 33 and 45% of the variability in cracking susceptibility. Microcracking only occurs on a strained cuticle. The sweet cherry CM is strained due to the early cessation of fruit growth. As the fruit increases in surface area, the cuticle becomes ‘diluted’ over the larger surface. This explains why larger fruit had thinner cuticles (r = -0.48**). Interestingly, the relationship between mean microcracking and mean fruit size was not significant across the genotypes investigated (r = 0.02), but highly significant when correlating microcracking and fruit size within a given genotype on an individual fruit basis (r = 0.32***). This indicates that factors other than the deposition pattern of cuticle are involved in microcracking and that these must differ between susceptible and tolerant genotypes. At present it is not clear what these factors are. Potential candidates include (1) the extent of cell wall swelling that might cause microcracking in the cuticle particularly above anticlinal cell walls, (2) the wetting characteristics of the fruit surface that may be affected by the uniformity of coverage of epicuticular wax, and/or (3) the chemical composition and rheological properties of epidermal and hypodermal cell walls. Further research on these important traits is needed to identify the factors involved in microcracking of the cuticle.

The relationship between cracking susceptibility and cuticle properties was weak. The variability in cracking susceptibility accounted for ranged from 12 to 18% as indexed by the partial r^2^ for the laboratory assessment. This is similar to our earlier study, where about 7% of published cracking susceptibility of 29 sweet cherry cultivars was accounted for by their cuticle mass per unit area Peschel and Knoche [51]. Two explanations may be offered for the higher partial r^2^ in the present study. First, the cracking susceptibility was assessed on the same batch of fruit as that used for isolating cuticles. Second, the genotypes used in the present study were full siblings, whereas those used in the study by Peschel and Knoche [51] were 29 unrelated commercial cultivars. It is interesting to note that it was the dewaxed cuticle (DCM) and the strain release that the statistical analysis identified as the variable with a highest predictive power for cracking among the cuticle characteristics. The DCM represents the cutin fraction which is a polymer network. In contrast to the monomeric wax fraction, the cutin network represents the structurally relevant part of the CM. That strain release was identified as a predictor is probably due to the positive relationship between cuticle strain and microcracking [1]. Clearly, CM mass, DCM mass, wax mass and strain relaxation upon isolation are all interrelated making it difficult to draw unifactorial conclusions.

The correlations of cracking susceptibility with the fruit Ca content were generally weak. The only significant relationship was between cracking susceptibility and the Ca/dry mass ratio in the stylar end region. Several factors account for this finding. First, stylar end cracking accounts for most cracking observed in the field [33, 34] and also for most microcracking [17]. Second, there is a significant gradient in the Ca/dry mass ratio and the osmotic potential from the stem cavity region to the stylar end region in this and our earlies study [52]. These gradients result from a successive break down of the xylem in developing sweet cherry fruit [47–49, 53]. Third, a higher Ca/dry mass ratio does not necessarily imply a lower cracking susceptibility as numerous studies on the effect of Ca spray application on fruit cracking have shown [46]. Recent findings indicate that the mode of action of Ca is primarily by preventing crack extension and that this requires the Ca to come into direct contact with the cell walls exposed in a microcrack [54]. This was not the case in our study.

### Potential adaptations of the phenotyping protocol for screening of larger number of genotypes

The logical next steps in research for a systematic improvement of cracking susceptibility of sweet cherry genotypes are QTL studies on traits that account for the differential susceptibility of genotypes to cracking. However, QTL studies require more genotypes than the number phenotyped in the present study. Unfortunately, a serious frost prevented this to be done in the growing season of the present study. Furthermore, for phenotyping a larger number of more genotypes, the protocol used herein should be simplified.

An important question arising is the following: Is an evaluation of cracking susceptibility needed in the year the phenotyping is performed? Based on the present study, the answer is probably Yes. Cracking susceptibility in the immersion assay determined using fruit from the same batch was predicted with higher coefficient of determination than cracking susceptibility rated in previous seasons under field conditions. Interestingly, the variables accounting for the two estimates of cracking susceptibility were identical (microcracking of the cuticle) or closely related (DCM mass per unit area, strain relaxation, Ca/dry mass ratio of the stylar end). Clearly, immersion assays during the harvest season tie up significant labor when time is scarce. However, our data reveal that a single 2 h or 4 h incubation interval would be equally well suited as an entire cracking index determination. The predictive power of a single determination at 2 or 4 h is identical to that of a cracking index at a marked reduction in the amount of labor. From a logistical point of view a 4 h incubation interval is advantageous.

Of the traits investigated, microcracking of the cuticle had the largest predictive power for cracking susceptibility–unfortunately, its quantification is laborious. In the present study microcracking was quantified twice: Immediately after harvest (‘before’) which reflected microcracking that resulted from the non-standardized natural exposure of fruit on the tree to rain and dew in the field. The second determination was on the very same fruit (detached) after a standardized 2 h incubation period in deionized water in the lab. The purpose of the incubation was to induce microcracks under standardized conditions. Since determinations were carried out on the same fruit, the increase in microcracking during the induction period could be calculated. Interestingly, microcracking before the induction and that induced during the induction were very closely related. Hence, susceptibility to microcracking resulting from natural rain did not differ from that during a standardized incubation procedure. This conclusion is likely to depend on the amount of precipitation that is typically encountered during the growing period of fruit which in turn will depend on the site where the fruit is grown. Our evaluations were carried out on fruit that received a significant amount of rain during the growing season.

In regions where precipitation is scarce, the microcracking in the field may be less and here, the 2 h incubation period for induction of microcracking would be needed. For regions with frequent and ‘reliable’ rain fall, a single evaluation immediately after harvest is likely to be sufficient. It is important to also note that the amount of labor required at harvest time for quantifying microcracking can be somewhat reduced by off-season image analysis. There is no need to inspect other regions of the fruit surface, since microcracking is most severe in the stylar end region, where wetness duration is typically long and the Ca/dry mass ratio low. From a technical point of view, assessing microcracking requires a dark room with a fluorescence dissecting microscope that is equipped with a digital camera and a filter set for viewing GFP fluorescence. When taking images, magnification and exposure times must be standardized.

Traits related to cuticle mass require an enzymatic isolation of the cuticle. Visual inspections by microscopy yield artefacts (overestimations) due to the strain relaxation of skin segments and cuticle. In addition, not more than a single ES per fruit must be excised. To avoid fracturing of the cuticle, the ES should be taken from the cheek where microcracking is typically less than in the stylar region [17]. In terms of equipment, disposable biopsy punches are preferred over old school cork borers because of their sharp blades and their precise diameters. This minimizes mechanical stress and strain during punching. Due to the strain relaxation on excision not more than one disc per fruit should be excised. Also, the fruit used for strain analysis must have representative size. The subsequent enzymatic isolation follows classical recipe [40]. For isolation technical grade pectinase and cellulase that is used for the clearing of fruit juice are sufficient and far less expensive than laboratory grade products offered by the usual chemical suppliers. For weighing a high-resolution microbalance is advantageous, but lack of resolution may be compensated by a larger number of cuticle discs per replicate. Strain release should be quantified on hydrated cuticles.

The amount of variability in cracking susceptibility accounted for by the Ca/dry mass ratio in the stylar region was lowest. Also, the Ca/dry mass ratio was only included in the regression model that explained the field cracking observations and not the lab cracking. Therefore, this trait should be given a low priority compared to microcracking and DCM mass per unit area. Further, a significant amount of seasonal variability may be expected for the Ca content of sweet cherries, since the Ca/dry mass ratio is markedly affected by the transpiration history of the fruit which in turn will reflect the water vapor pressure deficit during early fruit development [46, 47, 52].

In conclusion using this abbreviated phenotyping scheme, a larger population of genotypes can now be phenotyped for QTLs for microcracking and DCM mass per unit area. These two characteristics account for most of the crack susceptibility observed in the field and in the lab as would be expected based on the Zipper model.

## Supporting information

S1 Dataset The raw data of all figures and the data on mean fruit mass of the individual genotypes are available in the S1 Dataset. (XLSX)
